# Mucins and Pancreatic Cancer

**DOI:** 10.3390/cancers2041794

**Published:** 2010-10-25

**Authors:** Nicolas Jonckheere, Nicolas Skrypek, Isabelle Van Seuningen

**Affiliations:** INSERM, U837, Jean-Pierre Aubert Research Center, Team 5 "Mucins, epithelial differentiation and carcinogenesis", Lille, France; E-Mails: nicolas.jonckheere@inserm.fr (N.J.); nicolas.skrypek@inserm.fr (N.S.)

**Keywords:** pancreatic cancer, mucin, regulation, biomarker, therapy

## Abstract

Pancreatic cancer is characterized by an often dramatic outcome (five year survival < 5%) related to a late diagnosis and a lack of efficient therapy. Therefore, clinicians desperately need new biomarkers and new therapeutic tools to develop new efficient therapies. Mucins belong to an ever increasing family of *O*-glycoproteins. Secreted mucins are the main component of mucus protecting the epithelia whereas membrane-bound mucins are thought to play important biological roles in cell-cell and cell-matrix interactions, in cell signaling and in modulating biological properties of cancer cells. In this review, we will focus on the altered expression pattern of mucins in pancreatic cancer, from the early neoplastic lesion Pancreatic Intraepithelial Neoplasia (PanIN) to invasive pancreatic carcinomas, and the molecular mechanisms (including genetic and epigenetic regulation) and signaling pathways known to control their expression. Moreover, we will discuss the recent advances about the biology of both secreted and membrane-bound mucins and their key roles in pancreatic carcinogenesis and resistance to therapy. Finally, we will discuss exciting opportunities that mucins offer as potential therapeutic targets in pancreatic cancer.

## 1. Pancreatic Cancer

Pancreatic Ductal Adenocarcinoma (PDAC) is the fourth leading cause of death by cancer in the world. The survival rate is extremely low (3–5%) and the survival curve at five years is very short (six months) [1]. This dramatic outcome is related to a lack of efficient therapeutic tools and early diagnostic markers [2]. At the time of diagnosis, more than 80% of PDACs are already metastatic or locally advanced. Therefore, only about 10% to 15% of patients are considered eligible for surgical resection. For the majority of patients, palliative therapy with gemcitabine-based chemotherapy remains the only option. However, chemotherapeutic drug resistance is a common feature of pancreatic tumors. Chemotherapy produces benefits and symptom improvements in only 20–30% of patients. Gemcitabine resistance is not yet understood [3,4]. Understanding the mechanisms of that resistance may thus help overcome drug resistance and allow development of novel therapeutic approaches.

**Figure 1:**
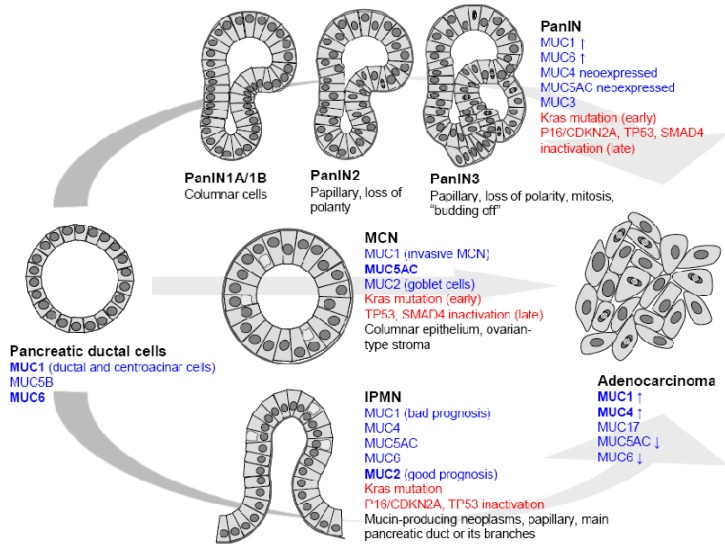
Mucin expression in precursor lesions leading to pancreatic ductal adenocarcinoma. Pancreatic intraepithelial neoplasia (PanIN), intraductal papillary mucinous neoplasm (IPMN) and mucinous cystic neoplasm (MCN).

Clinical, pathological and genetics studies have identified three different preneoplastic lesions of the duct as precursors of pancreatic ductal adenocarcinoma (PDAC) [5,6]. These lesions are pancreatic intraepithelial neoplasia (PanIN), intraductal papillary mucinous neoplasm (IPMN) and mucinous cystic neoplasm (MCN). Among these lesions PanIN are the most frequent and best characterized both at the morphological and molecular levels (Figure 1).

Pancreatic carcinogenesis follows a hyperplasia/dysplasia/*in situ* carcinoma/invasive carcinoma progression. Hruban and colleagues have proposed a model with initial ductal proliferation toward invasive ductal carcinoma. 85% of PDAC develop from PanIN in which histologic, cytologic and genetic alterations (K-ras mutation, p16/CDKN2A, Smad4 deletion) are accumulated [6]. PanIN1A and PanIN1B are characterized by hyperplasia without dysplasia. PanIN2 lesions show variable dysplasia and PanIN3 represent *in situ* carcinoma. As an early event leading to PDAC, PanIN and genes expressed in PanIN are therefore potential therapeutic targets (Figure 1).

Intraductal Papillary Mucinous Neoplasms (IPMN) are thought to be another precursor of PDAC. They originate in the main pancreatic duct and are characterized by a massive dilatation of the ducts or cyst formation and hypersecretion of mucins. Usually, IPMN have a good clinical course but 10 to 20% of IPMN are invasive carcinomas with bad prognosis. The mutational spectrum of IPMN slightly differs from PDAC since Smad4 mutations are relatively uncommon. The overall five year survival for surgically resected patients with invasive adenocarcinoma arising from IPMN is 45% [5].

Mucinous cystic neoplasms (MCN) are defined as mucin-producing cyst forming epithelial neoplasms of the pancreas with a distinct ovarian type-stroma. Cysts of MCN are lined by a columnar mucin producing epithelium which can have a broad spectrum of dysplasia. Activation of Kras is an early event in the development of MCN whereas *p53* and *Smad4* gene mutation occurs at a later stage. Usually, MCN have a good prognosis since the five year survival is close to 100% for patients who undergo surgical resection without invasive carcinoma. When associated with invasive cancer, the mean five year survival is 50–60% [5].

Pancreatic endocrine carcinoma (PECA) or endocrine tumors account for less than 2% of pancreatic malignancies, although infrequent, their incidence has been increasing. Surgery is the only treatment modality with the potential to cure, but it is only likely to be effective in patients without diffuse metastatic disease [7].

These disastrous statistics for pancreatic cancer show that it is urgent to develop new diagnostic and/or prognostic tools for the clinicians in order to propose better healthcare management of the disease. Moreover, identification of new molecular targets by deciphering the molecular mechanisms underlying the disease will allow the development of new therapeutical approaches to treat the disease.

## 2. Mucins

Mucins belong to a heterogeneous family of large *O*-glycoproteins composed of a long peptidic chain called apomucin on which are linked hundreds of oligosaccharide side chains representing 50–80% of the total molecular weight (MW) of the molecules. Mucins were initially described as secreted by epithelia, able to form viscoelastic gels and responsible for rheological properties of mucus [8].

The family of secreted mucins, gel-forming components of viscoelastic mucus gels protecting the epithelia, includes mucins MUC2, MUC5AC, MUC5B, MUC6, and MUC19. They are all structurally related to the superfamily of cystine-knot growth factors [9]. Their main function is to participate in mucus formation by forming a tridimensional network via oligomerization domains to protect underlying epithelia against various injuries (inflammation, bacteria, virus, pollutants, pH, *etc*). MUC7 and MUC9 are smaller secreted mucins that do not oligomerize. Interestingly, four of the genes encoding gel-forming mucins, MUC2-MUC5AC-MUC5B-MUC6, are clustered on the p15 arm of chromosome 11. Among the secreted mucins, Yamazoe *et al.* showed that MUC5AC might have the potential to accelerate progression of PDAC since MUC5AC inhibition markedly reduced invasive and adhesive activities of SW1990 pancreatic cancer cells [10].

Membrane-bound mucins, which contain a transmembrane (TM) domain, include MUC1, MUC3A/B, MUC4, MUC12, MUC13, MUC15, MUC16, MUC17, MUC20 and MUC21 [11]. Among them, MUC1 and MUC4 are best characterized and are described in Section 2.1, Section 2.2 below, respectively. Based on their structure and localization they are thought to play important biological roles in cell-cell and cell-extracellular matrix interactions, in cell signaling and in biological properties of cancer cells [12]. Moreover, because of their specific pattern of expression during the different steps of tumor progression toward carcinoma, membrane-bound mucins remain under intense investigation as both potent new biomarkers and therapeutic targets in epithelial cancers [12,13].

### 2.1. MUC1

MUC1 was the first mucin gene characterized [14,15,16]. The gene encodes a large apomucin lacking EGF domains usually found in the membrane-bound mucins. The MUC1 mucin is also known as polymorphic epithelial mucin (PEM), episialin, DUPAN-2, DF3, human milk fat globule (HMFG), epithelial membrane antigen (EMA) and CD227. The MUC1 apomucin contains an *O*-glycosylated repeated central domain and a 72 amino acid cytoplasmic tail. Numerous reports have shown that the cytoplasmic tail of MUC1 which contains a SH2 interaction domain and several kinase phosphorylation sites, acts as a docking protein for cell signaling involving notably β-catenin, c-Src, glycogen-synthase kinase-3β (GSK-3β), protein kinase Cδ (PKCδ), and the four members of the ErbB receptor family. These interactions induce signaling pathways such as the "mitogen-activated protein kinase" (MAPK) [12,17].

MUC1 plays roles in cell signaling, differentiation and proliferation of epithelial cells [18]. The biological properties of MUC1, its altered expression and post-translational modifications also confer an important role to MUC1 in tumor progression and metastasis [19,20]. Notably, down-regulation of MUC1 expression using RNA interference decreases the metastatic potential of pancreatic adenocarcinoma *in vivo* [21].

### 2.2. MUC4

MUC4 gene was discovered and characterized in our laboratory [22,23,24]. The gene encodes a large apomucin composed of two sub-units MUC4α and MUC4β. MUC4α is an extracellular subunit featuring a typical hyperglycosylated region. MUC4β is a membrane-bound sub-unit containing epidermal growth factor (EGF)-like domains. Experimental evidence suggests that the EGF-like domains play a role in receptor-ligand interactions. The interaction of the MUC4 EGF_1_-like motif with ErbB2 is the best characterized, likely serving as a regulator in signaling related to growth, motility or differentiation properties of the cell [12,25]. Rat Muc4 and ErbB2 interaction leads to signaling pathway modulation. Muc4-ErbB2 complex induces differentiation via activation of the cell cycle inhibitor p27kip, whereas formation of a quaternary complex of Muc4-ErbB2-ErbB3-neuregulin activates PKB/Akt and MAPK pathways leading to proliferation and inhibition of apoptosis [26]. From these data, Carraway and colleagues have proposed Muc4 as a modulator of proliferation and differentiation [27].

MUC4 may also play important roles in the behavior of epithelial tumor cells. Indeed, invalidation of MUC4 expression by integration of MUC4 antisense RNA (siRNA) leads to reduced proliferation, motility and increased cellular aggregation possibly via modulation of ErbB2 expression [28]. Stable ectopic expression of a truncated form of MUC4 (lacking 90% of the tandem repeat (TR) region), resulted in increased growth, motility, and invasiveness of pancreatic cancer cells and in xenografted nude mice [29].

## 3. Mucins and the Normal Pancreas

MUC1 is the main membrane-bound mucin expressed in the normal pancreas (Figure 1). The labeling concerns acinar cells at the cytoplasmic level, and ductal cells at the apical level [30]. Therefore, MUC1 has been used as a marker of pancreatic ductal cells in several studies [31,32].

Despite controversial initial results, no *MUC4* transcript or apomucin are found in normal pancreatic tissues [30]. However a number of non neoplastic duct lesions such as atrophic ducts, or ducts in the setting of an inflammatory reaction, show expression of MUC4.

Regarding secreted mucins, MUC2 is either absent or weakly expressed (33%) in ductal and acinar cells of the healthy pancreas [30,33]. *MUC5B* and *MUC6* mRNA have been observed in intralobular ducts by *in situ* hybridization (ISH). In immunohistochemistry studies, MUC5B mucin is found expressed in both acinar (33%) and ductal cells (80%). Similarly, 12–27% of pancreatic ducts express MUC6 mucin. MUC5AC is rarely expressed in pancreatic ducts (2–4%).

## 4. Mucins and Pancreatic Cancer

Pancreatic carcinogenesis is characterized by an altered pattern of mucin expression at different stages of tumor progression (Figure 1). Membrane-bound mucin production is detected as early as PanIN1A stage and is characterized by neoexpression of MUC4 and increase of MUC1. The PanIN3 stage, previously named *in situ* carcinoma, is characterized by a strong expression of MUC4 and MUC1 and occurrence of MUC3 [13,34]. During carcinogenesis, gradual expression of MUC4 has been demonstrated by IHC: 17% of PanIN1A, 36% of PanIN2 and 85% of PanIN3 express MUC4 [35]. In true PDAC, the prevalence of MUC4 apomucin expression reaches 83 to 89% [35,36]. These results are in agreement with gene expression profile studies which showed that MUC4 belongs to the most differentially expressed genes in PDAC [37]. The MUC1 mucin is more frequently expressed by PanIN lesions (around 70% of the cases) compared to normal pancreas. Around 80% of PDAC samples still express MUC1 [33]. MUC17 was also shown to be overexpressed in pancreatic tumor cell lines and tumor tissues compared with the normal pancreas [38]. So far, there is no information about MUC17 in PanIN early precursors. PanIN lesions and PDAC express gastric-type mucin (MUC5AC and MUC6) but do not express intestinal type mucin MUC2. MUC5AC which is not expressed in healthy pancreas is neoexpressed in earliest stage PanIN1A (70% of cases) and its expression reaches 85% in PDAC [33]. MUC6 increase in PanIN1A has also been reported. MUC6 expression peaks in PanIN1A (74%) and then decreases during carcinogenetic progression (35% of PDAC).

IPMN expressing MUC1 correspond to invasive carcinoma with a short survival rate and bad prognosis whereas those that express MUC5AC correspond to slow-growing adenoma with good prognosis. The majority of IPMN express MUC2 (76–85% of cases) [39,40]. MUC1 expression is predominantly observed in pancreatobiliary-type papillae (44%) whereas MUC2 is expressed uniformly and diffusely in intestinal-type papillae (92%) but rarely and focally in the pancreatobiliary type (19%) [41]. Interestingly, MUC2+ MUC1− group of tumors (56%) is associated with an intermediate behavior [40], and is opposed to the data of Nakamura *et al.* who showed that MUC2+ tumors are correlated with a higher potential of malignancy than MUC2- tumors [42].

Because MCN are rare, no consensus about mucin expression has been established. Luttges *et al.* have shown that non invasive MCN are characterized by the expression of MUC5AC and lack of MUC1 whereas MUC2 is restricted to goblet cells scattered within the MCN epithelium. Invasive forms of MCN are MUC1 positive [43]. On the contrary, Terada *et al.* have reported that MUC1, MUC2, MUC3, and MUC5AC/MUC6 (both recognized by the A-HF antibody) apomucins were expressed in 7/8, 0/8, 2/8, and 3/8 cases, respectively [44].

## 5. Mucin Gene Regulation in Pancreatic Cancer

A better understanding of the molecular structure of regulatory regions as well as mechanisms governing mucin expression is also mandatory if one wants to assign direct roles to mucins in carcinogenesis and better understand their influence on the biological properties of the tumor cell. Studies aiming at deciphering the signaling pathways will allow identification of potential therapeutic targets.

*MUC1* promoter has been extensively characterized mainly in breast cancer [13]. Despite being overexpressed in pancreatic cancer, no specific pancreatic transcriptional regulation has been published.

The *MUC4* 5’-flanking region (GenBank accession number: AF241535) has been characterized and contains two active promoters. A TATA-less proximal promoter, mainly composed of GC-rich domains that bind the transcription factor Sp1 as well as the CACCC box binding protein and a very high density of binding sites for factors known to initiate transcription in TATA-less promoter (Sp1, CACCC box, glucocorticoid receptor element, AP-1, polyomavirus Enhancer Activator-3 (PEA3) and Med-1). The distal promoter is characterized by a TATA box and contains numerous putative binding sites for Sp1, AP-1, AP-4, GATA and cyclic adenosine monophosphate (cAMP) responsive element binding protein (CREB) transcription factors [13,45].

Pancreatic cancer cells have been the major model for *MUC4* regulation. In that model, *MUC4* was shown to be regulated by a wide range of specific factors. Tumor suppressor AP-2 down-regulates MUC4 expression at both protein and transcriptional levels *via* two AP-2 *cis*-elements located in the proximal promoter [36]. Interestingly, AP2-α overexpression also leads to an increase of gemcitabine sensitivity [46]. The Ets family member PEA3 is implicated in a wide range of cellular processes such as differentiation, proliferation and transformation [47] and was shown to upregulate *MUC4* expression in synergy with c-Jun and Sp1 whereas PEA3 represses the transcriptional activity of ErbB2 promoter [48]. IFN-γ inflammatory pathway increases *MUC4* expression via STAT-1 upregulation [45,49].

In pancreatic carcinogenesis, TGF-β, which possesses both tumor-suppressive and oncogenic activities [50], is a strong activator of MUC4 expression [51,52]. TGF-β regulation involves a cooperation between Smad2 and Smad4 transcription factors, and Smad4 binding to seven Smad Binding Elements (SBE) located along proximal and distal promoters. When Smad4 is mutated and inactive as observed in 50% of PDAC, TGF-β_1_ is still able to activate MUC4 expression *via* mitogen activated protein kinase (MAPK), phosphoinositide-3 kinase (PI3K) and Protein Kinase A (PKA) signaling pathways [52]. Moreover, all-trans-retinoic acid (RA) treatment increases MUC4 expression via retinoic acid receptor-α (RAR-α). That upregulation is a consequence of the increase of expression of TGF-β_2_ regulating *MUC4* expression in an autocrine manner [51]. IFN-γ and RA can synergize at the transcriptional level to affect *MUC4* induction in pancreatic tumour cells [53]. *MUC4* expression is negatively regulated by the “cystic fibrosis transmembrane conductance regulator” (CFTR) chloride channel that is implicated in multiple cellular functions. CFTR mutation carrier status is correlated with early-onset of pancreatic adenocarcinoma [54]. It was observed that CFTR was downregulated in pancreatic cancer cells and negatively correlated with MUC4 [55].

Very little is known about the regulation of the other membrane-bound mucins in pancreatic cancer. Only a strong enhancer was found in the *MUC17* upstream region with a significant promoter activity in AsPC-1, and HPAF pancreatic cancer cell lines [38].

Despite an extremely intriguing upregulation in the earliest stage of pancreatic carcinogenesis, mechanisms that control atypically expressed secreted mucins remain to be deciphered. Transcriptional activity of the *MUC5AC* promoter region was analyzed in pancreatic cancer cell lines, SW1990 and HPAF. Sp1 and AP-1 were identified as key factors responsible for basal and PMA-induced *MUC5AC* transcription. PMA-induced upregulation is mediated by Sp1, PKC/ERK/AP-1 and PKC/JNK/AP-1 pathways [56]. Moreover, stimulation of adenylyl cyclase and the PKA pathway by forskolin and vasoactive intestinal peptide (VIP) increased MUC5AC antigen expression and release from pancreatic cancer cells suggesting that PKA may contribute to the up-regulation of MUC5AC expression seen in PanIN1A [57]. cDNA microarray analysis has enlightened the hypothesis that upregulation of gastric-type mucins (MUC5AC and MUC6) could be related, in a similar manner to the majority of foregut markers seen in early PanIN lesions, to the Hedgehog-mediated conversion of the normal pancreatic epithelium to a gastric epithelial differentiation program [58].

The correlation between mucins and foetal pancreatic development, that links the patterns of mucin expression found in PDAC, is not proved at this time. *In vitro* studies have shown so far that hepatocyte nuclear factors (HNF-1/-4), forkhead box A (FOXA1/A2), GATA-4/-5/-6, and caudal-related homeobox (CDX-1/-2) transcription factors which control cell differentiation of gut endoderm derived-tissues (including pancreas) during embryonic development, are potent regulators of human *MUC2*, human *MUC4* and murine *Muc5ac* mucin genes [59,60,61,62]. Therefore, differentiation network involved in pancreatic development could also regulate mucin expression in a similar manner during carcinogenesis.

## 6. Epigenetic Regulation of Mucin Genes in Pancreatic Cancer

The term “epigenetics” refers to a heritable change in gene expression that is driven by mechanisms other than alteration of the nucleotide sequence [63]. Epigenetic changes may occur either at the transcriptional (methylation of DNA, methylation, acetylation and phosphorylation of histones) or post-transcriptional (miRNA) levels. These mechanisms are often altered in pathological processes [64]. Alterations of expression include both silencing and overexpression of mucin genes while promoters of mucin genes share a GC-rich structure and, for most of them, contain a CpG island, two structures characteristics in favor of epigenetic regulation [65,66].

*MUC1* gene expression is regulated by a tightly related combination of DNA methylation and histone modification via histone H3 lysine 9 (H3-K9) in the 5'-flanking region of the *MUC1* promoter in cancer cell lines including HPAFII (MUC1+), BxPC3 (MUC1+), PANC1 (MUC1+/–) pancreatic cell lines [67].

*MUC4* proximal and distal promoters, which possess each a CpG island, are heavily methylated whether the gene is expressed or not in the cells. On the contrary, *MUC4* 5’-untranslated region (UTR) show a methylation profile correlated to the level of MUC4 expression in the cells. Moreover, this hypermethylation is correlated with a repressive histone code including histone deacetylation via HDAC3 and methylation of histone H3 via DNA methyltransferase (DNMT)-3A and DNMT3B [68]. Yamada and collaborators later confirmed the CpG methylation of *MUC4* 5’-UTR using the MassARRAY® compact system [69].

*MUC2*, *MUC5AC*, *MUC5B* and *MUC6* genes belong to the 11p15 mucin gene cluster, which is located in a hot spot of abnormal methylation in cancer [70]. Epigenetic regulation of genes encoding secreted mucins has been mainly described in other tissues than pancreas [66].

Expression of *MUC2* mRNA has been correlated with methylation of the proximal region of the promoter in pancreatic cancer cells whereas no direct link was found between methylation and *MUC5AC* expression. [71]. In PANC-1 cells, *MUC2* expression is induced by 5-aza 2’-deoxycytidine (5-aza) and histone deacetylation. In a similar manner, 5-aza treatment leads to *MUC5B* increased mRNA level in CAPAN-1 pancreatic cancer cells. Chromatin immunoprecipitation (ChIP) assays indicated that, in PANC-1 cells, *MUC2* and *MUC5B* repression was associated with histone H3 deacetylation and K9H3 methylation as well as with K27H3 trimethylation for *MUC5B* [72].

Recently, methylated CpG mapping of *MUC2* and *MUC5AC* promoters have been determined in pancreatic cancer cell lines [73,74]. It was shown that promoter methylation acts in combination with associated H3 histone deacetylation [74,75].

So far, no data is in favor of MUC6 epigenetic regulation in pancreatic cancer.

miRNAs are differentially expressed during pancreatic carcinogenesis as in numerous organs. Interrogation of MiRbase and MiRanda databases revealed a large number of miRNAs that could potentially target the 3′-UTR of mucin genes including *MUC1*, *MUC4*, *MUC17* or *MUC2*, *MUC5AC*, and *MUC6* [66]. Very recent data indeed indicate that the *MUC1* 3’-UTR is directly regulated by miR-125b, miR-145 and miR-1226 in human breast cancer cell line [76,77,78].

## 7. Mucins as Biomarkers of Pancreatic Cancer

A major issue with PDAC is the dramatic lack of efficient biomarkers. Serum-based molecular markers include carbohydrate antigens CA 19-9 (sialyl Lewis blood antigen), CA125, and SC6-Ag, pyruvate kinase isoenzyme type 2 (M2-PK) and secreted proteins such as macrophage inhibitory cytokine 1 (MIC-1) [79]. Among available markers, those with a good sensitivity are also positive in patients with benign diseases and thus cannot be used routinely as screening or diagnostic markers. They still lack sensitivity, specificity or reproducibility. In clinical practice, the most commonly accepted use of CA 19-9 is to assess the prognosis and monitor the response to therapy.

Aberrant profiles of mucin expression offer promising options as biomarkers (see section 4). Notably, detection of MUC4 mucin could help distinguish reactive ductal epithelial cells from the cells of pancreatic adenocarcinoma in fine needle aspirate (FNA) samples guided by endoscopic ultrasound (EUS) [80]. Furthermore, the epitope born by the anti-MUC1 antibody (PAM4) is neoexpressed in early lesions and PDAC [81].

Generating multiple markers in combination with mucin typical pattern of expression may also provide a new approach for early detection of pancreatic cancer. For example, the detection of a glycan variant on MUC5AC mucin, using the lectin wheat-germ agglutinin, allows discrimination between MCN and IPMN from benign cystic lesions when used in combination with cyst fluid CA 19-9 with a better sensitivity and sensibility. These biomarkers were more efficient than cyst fluid carcinoembryonic antigen (CEA) [82].

## 8. Mucins as Therapeutic Tools in Pancreatic Carcinogenesis?

**Figure 2:**
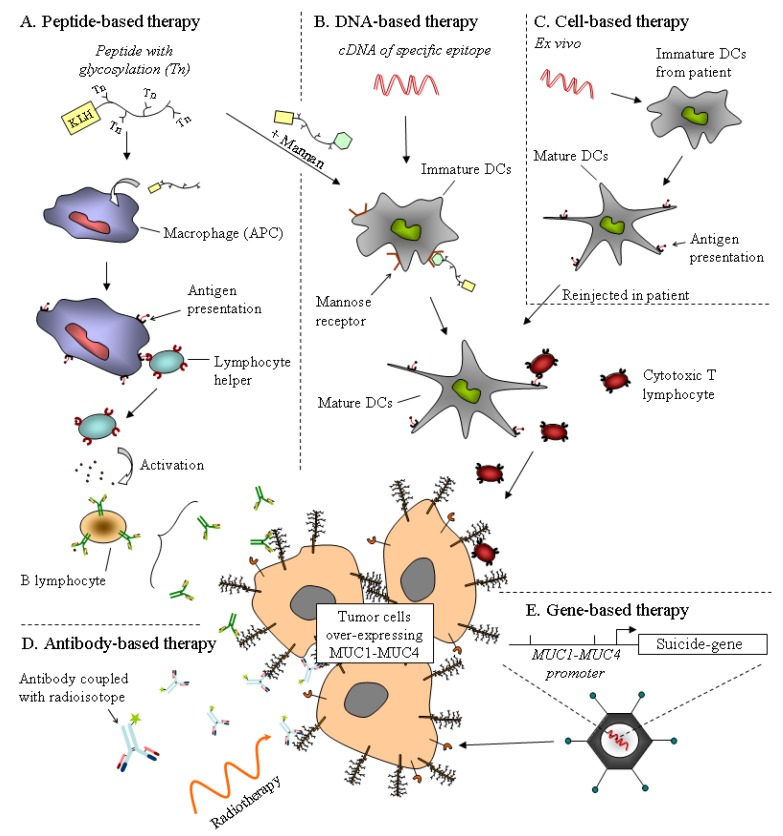
Strategies for mucin-based therapies already used in phase trial. The aim of antigen-based therapy, by the use of a peptide or a cDNA derived from mucin epitopes, is to activate the immune system against tumor cells overexpressing the mucin. (**A**) The glycosylated mucin peptide, with KLH adjuvant, activates the humoral response by the activation of antigen-presenting cells (APC). The efficiency of this peptide may be increased with a mannan epitope recognized by a mannose receptor on dendritic cells (DCs), which in turn activates the cytotoxic response. (**B**) DNA vaccine encoding a specific mucin epitope is incorporated by DCs and activates the cytotoxic T lymphocyte. (**C**) Cell-based therapy is an alternative of DNA vaccine. Immature DCs extracted from the patient are incubated with DNA encoding the mucin epitope. Activated DCs exhibiting mucin antigen are injected into the patient. (**D**) Antibody-based therapy. Use of antibodies is promising for the detection and treatment of cancer. Antibodies against mucin epitopes are coupled with a radioisotope for radiotherapy ((131)I-PAM4) or a cytotoxic drug to target tumors. (**E**) Gene-based therapy. Promoter of mucin may be used to deliver a cytotoxic agent on tumor cells.

Mucins are thought to play important roles in the biological properties of tumor cells. One could consider mucins as promising therapeutic tools for gene therapy and immunotherapeutic approaches. Lack of effective therapeutic tools is a major reason for the dramatic outcome of PDAC. Using mucins as therapeutic targets could be a way to either potentialize existing therapy or develop new treatment for PDAC. So far, immuno-, gene based- and chemotherapy using mucins as therapeutic tools have been developed and hold promise (Figure 2).

### 8.1. Immunotherapy

MUC1 peptide core or glycopeptides have been used in immunotherapy assays mainly in breast cancer [12,83,84] that could be potentially useful in PDAC. Mab-PAM4 has been recently proposed as a promising anti-MUC1 antibody which reacts with 85% of pancreatic adenocarcinomas while showing no reactivity against normal pancreas or other tissues. Immuno- and radio-therapy can be combined to increase efficiency and tumor response. Combined chemotherapy with gemcitabine and low dose ^(131)^I-PAM4 radioimmunotherapy may provide an improved alternative for the treatment of pancreatic cancer [81].

Recently, a peptide-based therapy using MUC1 protein transduction domain PTD4 (PMIP), which targets MUC1, EGFR and β-catenin interaction, displayed great efficiency in xenografted and transgenic mice models and inhibits cancer progression. This indicates a potential clinical implication in the treatment of MUC1+ pancreatic cancer [85].

### 8.2. Gene-Based Therapy

Promoters of mucin genes could also be used to drive cytotoxic agents (suicide genes) into the cells. Adenovirus with *MUC1* promoter driving human somatostatin receptor subtype 2 (hSSTR2) in PANC-1 pancreatic cancer cells demonstrates a significant inhibition of cell proliferation in MUC1+ pancreatic carcinoma [86]. Since MUC4 is neoexpressed in early PanIN, its promoter could be used in a similar manner to target early pancreatic lesion.

Preliminary work has been conducted using MUC4 as a therapeutic target in DNA vaccine assays. Dendritic cell (DC)-based vaccine using MUC4 generated potent cytotoxic responses suggesting that PACRE/MUC4 DNA vaccine could also be a potential strategy for immunotherapy of MUC4+ tumors [87].

### 8.3. Chemotherapy

Gemcitabine is a 2′,2′-difluoro-2′-deoxycytidine analog which bioactivity results in cell apoptosis. For the majority of PDAC patients, resistance to gemcitabine-based chemotherapy is a common feature. Thus, chemotherapy produces benefits and symptom improvements only in 20–30% of patients. Understanding these mechanisms may help overcome drug resistance. MUC4 invalidation blocks activation of intrinsic apoptosis pathway induced by gemcitabine treatment. MUC4 promotes sequestration of Bad proteins in the cytoplasm and deactivation of Bcl_XL_ via MUC4-ErbB2-ERK mediated pathway and therefore might contribute to gemcitabine resistance of total population and CD133^+^ cells [88,89]. Kalra and collaborators also showed that in pancreatic tumor cells, *O*-glycosylation inhibitor benzyl-α-GalNAc promotes the effectiveness of 5-fluorouracil (5-FU) [90]. Thus, intracellular uptake, antineoplastic and antitumor drug effects of 5-FU is enhanced in a reduced extracellular matrix environment with limited amount of mucins induced by intratumoural (IT) injections of benzyl-alpha-GalNAc [91].

## 9. Perspectives

So far, evaluation of the biological roles of mucins in pancreatic cancer has been carried out *in vitro* using cancer cell lines or *in vivo* using xenograft approaches. The development of new animal models by generating specific knockout (KO) mice or transgenic mice overexpressing mucins should provide new information about their role *in vivo*.

Very few mouse models (Muc1/Muc2/Muc16) have been published so far. MUC1 transgenic mice (MUC1.Tg), which express MUC1 in a similar pattern and level as observed in humans, have been crossed with LStopL-Kras^G12D^ pancreatic cancer developing mice. The presence of MUC1 in these transgenic mice enhanced PanIN progression and development of pancreatic adenocarcinoma via an immunosuppressive effect due to a higher level of cyclooxygenase-2 or indoleamine 2,3-dioxygenase [92].

Mouse models have previously provided evidence of a direct role of MUC1 in tumor progression in other pathological models despite the fact that Muc1 KO mice develop normally, are healthy and fertile. These mice, however, showed a significantly slower growth rate of primary breast tumors [93]. Using the MUC1.Tg model, Lalani *et al.* showed a reduction in tumor incidence at low inocula and a delay in tumor growth at higher inocula [94]. This indicated for the first time that Muc1 participates in tumor progression. As for MUC1, future mucin KO mice could be crossed with models such as LStopL-Kras^G12D^, Trp53R172H, Ink4α or mice deficient in TGF-β/Smad pathway components. These models develop spontaneously pancreatic adenocarcinoma and faithfully recapitulate the PanIN sequence and therefore provide outstanding pathological study models [95,96,97,98].

Muc2 deficient mice spontaneously develop colonic adenoma that may degenerate into colorectal tumors [99]. Muc2 is thus involved in the suppression of colorectal cancer but the question “cause or consequence” of Muc2 role in tumor suppression remains to be answered. A Muc2 deficient mouse could be combined with a murine model of IPMN that remains to be characterized in order to assess the direct role of this secreted mucin in IPMN development.

Finally, Muc16 homozygous mutant mice are viable, fertile, and develop normally [100]. This KO mouse model provides a unique platform for future studies to identify the role of CA125/MUC16 in ovarian cancer. However, the potential use for pancreatic cancer remains to be demonstrated.

In the future, development of mouse models for MUC4, MUC5AC and MUC6 mucins that are overexpressed during pancreatic carcinogenesis would be of an extremely valuable interest and would greatly help the scientific community demonstrate the potential of these mucins as therapeutic tools in this disastrous cancer.
